# Development and Evaluation of Nomograms to Predict the Cancer-Specific Mortality and Overall Mortality of Patients with Hepatocellular Carcinoma

**DOI:** 10.1155/2021/1658403

**Published:** 2021-03-29

**Authors:** Xiaofeng Ni, Ding Li, Shengjie Dai, Hao Pan, Hongwei Sun, Jianyang Ao, Lei Chen, Hongru Kong

**Affiliations:** ^1^Department of Surgery, The First Affiliated Hospital of Wenzhou Medical University, Wenzhou, Zhejiang Province, China; ^2^Key Laboratory of Diagnosis and Treatment of Severe Hepato-Pancreatic Diseases of Zhejiang Province, Zhejiang Provincial Top Key Discipline in Surgery, The First Affiliated Hospital of Wenzhou Medical University, Wenzhou, Zhejiang Province, China; ^3^Department of Orthopaedics, The First Affiliated Hospital of Wenzhou Medical University, Wenzhou, Zhejiang Province, China

## Abstract

Hepatocellular carcinoma (HCC) is the most common type among primary liver cancers (PLC). With its poor prognosis and survival rate, it is necessary for HCC patients to have a long-term follow-up. We believe that there are currently no relevant reports or literature about nomograms for predicting the cancer-specific mortality of HCC patients. Therefore, the primary goal of this study was to develop and evaluate nomograms to predict cancer-specific mortality and overall mortality. Data of 45,158 cases of HCC patients were collected from the Surveillance, Epidemiology, and End Results (SEER) program database between 2004 and 2013, which were then utilized to develop the nomograms. Finally, the performance of the nomograms was evaluated by the concordance index (C-index) and the area under the time-dependent receiver operating characteristic (ROC) curve (td-AUC). The categories selected to develop a nomogram for predicting cancer-specific mortality included marriage, insurance, radiotherapy, surgery, distant metastasis, lymphatic metastasis, tumor size, grade, sex, and the American Joint Committee on Cancer (AJCC) stage; while the marriage, radiotherapy, surgery, AJCC stage, grade, race, sex, and age were selected to develop a nomogram for predicting overall mortality. The C-indices for predicted 1-, 3-, and 5-year cancer-specific mortality were 0.792, 0.776, and 0.774; the AUC values for 1-, 3-, and 5-year cancer-specific mortality were 0.830, 0.830, and 0.830. The C-indices for predicted 1-, 3-, and 5-year overall mortality were 0.770, 0.755, and 0.752; AUC values for predicted 1-, 3-, and 5-year overall mortality were 0.820, 0.820, and 0.830. The results showed that the nomograms possessed good agreement compared with the observed outcomes. It could provide clinicians with a personalized predicted risk of death information to evaluate the potential changes of the disease-specific condition so that clinicians can adjust therapy options when combined with the actual condition of the patient, which is beneficial to patients.

## 1. Introduction

Hepatocellular carcinoma (HCC) is the most common type of primary liver cancers (PLC), which comprises 90% of all liver carcinomas, and ranks second as a reason for cancer-related mortality around the world [1, 2].

Numerous factors have been reported to be associated with the mortality and prognosis of HCC patients, including age and the tumor burden defined by the National Cancer Institute (NCI) that refers to the number of cancer cells, tumor size or the overall amount of cancer in the body [3], pathological grade, and presence of a metastasis [4]. With improvements in the diagnostic technology and therapy options, patients have received standardized therapy, which has significantly improved their quality of life [5]. As a malignant tumor, however, the 5-year survival rate of HCC has stayed in the range of 15%–40% due to its low early diagnosis rate and high recurrence and metastasis rates after resection [5, 6], which makes it a serious disease that affects people's health. Moreover, recent clinical practice guidelines from the European Association for the Study of the Liver (EASL) have pointed out the necessity for the stratification of the risks of HCC patients [1].

With the development of biomedical technology, some biomedical databases have emerged that are expected to support personalized medicine and provide effective management of humans [7]. The big data era can optimize medical management programs, providing better patient care and treatment, improving population health, and reducing costs [8]. In addition, machine learning algorithms based on big data can predict individual patient disease-specific risks and disease-specific mortality and identify which therapy will be precise and beneficial to patients [7].

The SEER database, supported by the Surveillance Research Program (SRP) in NCI's Division of Cancer Control and Population Sciences (DCCPS), is one of the most representative large-scale tumor registration databases that collects a large number of evidence-based medicine data and provides systematic evidence and valuable first-hand information for clinicians' evidence-based practice and clinical medical research. The clinical data provided by the SEER database includes the patient's registration number, personal information (i.e., age, sex, race, marriage, and insurance), primary lesion location, tumor size, treatment plan, and cause of death. Therefore, we collected a large amount of clinical characteristic data of HCC patients using this database, which is the basis for the development of our model.

Nomograms are a graphic description of a predictive model derived from personal predictive information that can be used to evaluate a numerical probability of events such as survival and mortality [4]. Ma et al. [9] developed a nomogram based on serum lncRNA to identify the biomarkers for diagnostic and treatment of HCC. Chen et al. [10] developed and verified a simple to use nomogram to predict the early survival of HCC for clinicians to promote communication with patients and the personalized evaluation after surgery. Lu et al. [11] explored a noninvasive method to construct fingerprint of preoperative plasma/nomogram to predict the recurrence risk of liver transplantation for HCC. They are also helpful for the clinician who can provide a visual interface to communicate with HCC patients [4]. To improve our nomogram's accuracy, a large amount of patient data is indispensable. Additionally, we utilized the calibration curve and an area under the ROC curve (AUC) to evaluate the accuracy and the predictive performance of the nomogram, as shown in recent studies completed by Le and Ou [12] to construct a predictive flavin adenine dinucleotide (FAD) binding sites and evaluate by the AUC to represent the predictive performance of this model. Other studies by Le et al. [13] introduced a useful tool to classify Rab protein by utilizing deep convolutional neural networks, and it was evaluated by ROC analysis which was usually used to measure for judging binary classifiers. In addition, Le et al. [14] evaluated the accuracy of the classifier called iEnhancer-5Step by utilizing the ROC curve to contrast the efficiency of different models.

The elderly has a lot of increased risk of death, and targeted interventions for HCC patients should be based on HCC-specific mortality. The competing risk analysis can be used to represent independent factors in the nomogram with different ranges of risk scores and then add these scores to obtain a total risk score to better achieve risk stratification, evaluation, and treatment options [15]. Compared to related nomograms reported in the recent years, we hoped to develop nomograms for long-term follow-up of HCC patients because of the poor prognosis and survival rate of HCC so that clinicians can identify the individual's risk of death and make adjustments to their current treatment accurately and beneficially.

## 2. Patients and Methods

### 2.1. Origin of the Patient Characteristic Data

A total of 45,158 cases of available providing patient information and clinical characteristics were obtained from the SEER database during the period of 2004 to 2013. The inclusion criteria were that patients diagnosed with HCC as the main diagnosis and excluded other malignant tumors were eligible for our study. A total of 24,647 patients who had completed follow-up for more than one year were identified; the follow-up was suspended when the HCC patient died or lost in contact. The SEER^∗^Stat software (Version 8.3.5, National Cancer Institute, Bethesda, MD, USA) was utilized to extract patient data with complete follow-up from the SEER database. No approval from the institutional review board was required because the SEER database is publicly available. The private data of all patients has been removed from the SEER database, and thus, no informed consent was required. All authors signed authorization that they had obtained permission from the SEER database to use its data.

### 2.2. The Arrangement of Patient Data

Our study cohort lists the characteristics of HCC patients and survivor characteristics at 1, 3, and 5 years since diagnosis. All values are presented as quantity and percentage of cases after sorting them out into baseline characteristics. The following categories were selected for our research: age, sex, pathological grade, the AJCC stage [16], surgery, radiotherapy, insurance, and marriage status. Because some of the HCC patients' information registered in the SEER database was incomplete, categories such as race, pathological grade, tumor size, the AJCC stage, and treatment options are listed separately. Univariable and multivariable analyses for overall survivals were performed to select variables in the predictable model and also competing risk analysis for cancer-specific survival prediction.

### 2.3. Calculation of Cumulative Incidence of Mortality

We calculated the cumulative incidence of the mortality for HCC mortality by the AJCC stage, surgery, and radiotherapy. When evaluating the CIF curve, the AJCC stage was divided into four groups including stages 1, 2, 3, and 4. Therapy options included surgery and radiotherapy. We divided the overall mortality into two groups including with and without therapy; four groups were utilized to evaluate cancer-specific and other-cause mortality. Then, the CIF curve was plotted. The *X*-axis represents the survival time, and the *Y*-axis represents the cumulative incidence of mortality. Each group is distinguished by the indicated different solid lines and dashed lines.

### 2.4. Development of the Nomograms

The nomograms for predicting cancer-specific mortality were constructed based on the Fine and Gray competing risks model [17], while the nomogram for predicting overall mortality was based on Fine-Gray (subdistribution hazard) model. Common variables in clinical practice including clinical characteristics (age, sex, race, pathological grade, tumor size, T category, N category, M category, and AJCC stage), therapy method (surgery and radiotherapy), and social status (insurance and marriage status) were included in the analysis. The highest alpha-fetoprotein (AFP) test results prior to treatment were also documented in the SEER database for HCC which could be important for prognostic prediction, but only recognized as “positive” or “negative” without exact lab values or a clearly stated standard to define “positive” and “negative.” Therefore, factor of AFP was excluded in this study. The univariable and multivariable analyses are aimed at identifying independent factors of HCC and represented in the nomogram. Related variables (included age, sex, race, pathological grade, AJCC stage, therapy options, and social status) with their *P* values less than 0.05 were selected to develop the final nomogram. Generally, every component in a nomogram was required to have a range of 0–100. The kernel of the nomogram was ensuring which component had the most significant influence on the predicted outcomes. In a word, the scale of every component of nomogram was constructed based on the most influential indicator. Then, we assigned a score according to the converted coefficient value. Finally, we graphically converted the model through the relevant code to form the nomogram by using R.

### 2.5. Evaluation of the Nomograms

To evaluate the nomograms, we plotted a calibration curve in order to assess the conformity graphically between the predicted outcome and observed outcome [18]. The value of the C-index ranges from 0 to 1, and the greater the value of the C-index over 0.5, the higher the predictive performance the nomogram possesses. In general, the C-index is 0.50-0.70 with low accuracy; between 0.71 and 0.90 is medium accuracy; greater than 0.90 is high accuracy. Intuitively, the ideal prediction of the calibration curve would present a 45-degree diagonal. In addition, we utilized the td-AUC as an indicator to evaluate the performance of the nomogram [19]. The AUC value provides a probability value ranging from 0.5 to 1. The greater the ROC curve deviates from the 45-degree diagonal, the more propinquity to the point (0, 1), and the greater the AUC value, the better the prediction performance.

### 2.6. Statistical Analysis

All data were statistically analyzed by utilizing the R software, version 3.5.1 (http://www.r-project.org/). R packages “regplot” (version 1.0), “mstate” (version 0.2.11), “survival” (version 2.44-1.1), “cmprsk” (version 2.2-9), “Hmisc” (version 4.2-0), “timeROC” (version 0.3), and “rmda” (version 1.6) were utilized to develop and verify the nomograms. All *P* values resulted from the use of two-sided statistical testing, and a probability less than 0.05 was considered statistically significant.

## 3. Results

### 3.1. Patient Baseline Characteristics

A total of 45,158 cases of eligible HCC patients screened from the SEER database between 2004 and 2013 were summarized by a series of patient and clinical characteristics as shown in Table 1. The following categories are listed in Table 1: age, sex, race, pathological grade, AJCC stage, surgery, and radiotherapy. Some of the categories were listed separately due to the lack of patient information.

In regard to the age composition of the whole cohort, the majority of patients (90.5%) were aged <80 years, and 9.5% of patients were aged ≥80 years. In regard to the sex and race composition as shown in Table 1, the majority of the patients were male (76.7%) and white (68.1%). Among the patients with specific pathological grades, there were 29.7% of patients in grade 1 or 2 and 8.4% of patients in grade 3 or 4, but 61.9% of patients had failed to be clearly graded. For the distribution of T stage, there were 0.1, 39.7, 21.4, 20.8, and 3.5% for the stages of T0, T1, T2, T3, and T4, respectively, and similarly, 14.6% of patients failed to be clearly T stage distributed. A total of 2737 patients (6.1%) were positive for lymph node involvement, and a total of 5568 patients (12.3%) presented with distant metastases. For distribution for the clinical stage according to the AJCC stage, patients in stages I, II, III, and IV accounted for 34.0, 18.0, 14.9, and 15.7%, respectively. In total, the patients treated with surgery accounted for 27.6%, and 6.3% of the patients who were treated with radiotherapy.

The median follow-up among the whole cohort was 10 months, with 25^th^ and 75^th^ percentiles ranging from 3 to 25 months. A total of 24,647 (54.6%) patients in the cohort had complete follow-up for at least one year. A total of 30940 (68.5%) patients died during the 5-year follow-up of this cohort.

### 3.2. Cumulative Incidence of HCC Mortality

**Figure 1:**
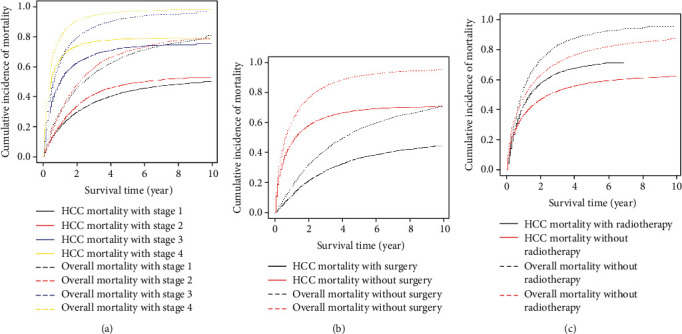
Cumulative incidence estimates of mortality according to patient and survivor characteristics. (a) The cumulative incidence of the overall mortality and HCC mortality of AJCC stage. (b) The cumulative incidence of the overall mortality and HCC mortality of surgery. (c) The cumulative incidence of the overall mortality and HCC mortality of radiotherapy.

The cumulative incidence function (CIF) curves are plotted in Figure 1, which present estimates of the cumulative incidence of mortality by AJCC stage, surgery, and radiotherapy. Survivor characteristics of the HCC patients since diagnosis at 1-, 3-, and 5-years are presented in Table 1. We observed from Figure 1(a) that both overall mortality and HCC mortality were significantly positively correlated with AJCC stage; the mortality of stages 3 and 4 was significantly higher than stages 1 and 2.  Figure 1(b) showed that the overall mortality of the surgical therapy group was significantly lower than nonsurgical therapy group, whereas there was no obvious discrimination between the overall mortality of the radiotherapy group and the nonradiotherapy group, especially in the early period since diagnosis (Figure 1(c)).

### 3.3. Univariable and Multivariable Analyses of Cancer-Specific and Overall Survival

Univariable and multivariable analyses of cancer-specific survival are shown in Table 2, while the univariable and multivariable analyses of overall survival are shown in Table 3. From the competing risk analysis of cancer-specific survival, male of sex (*P* = 0.002, SHR = 1.132, and 95% CI 1.046-1.225), high level of grade (*P* < 0.001, SHR = 1.757, and 95% CI 1.631-1.892), tumor size greater than 5 mm (*P* < 0.001, SHR = 1.577, and 95% CI 1.459-1.705), lymphatic metastasis (*P* < 0.001, SHR = 1.220, and 95% CI 1.087-1.368), distant metastasis (*P* < 0.001, SHR = 1.550, and 95% CI 1.398-1.718), and advanced AJCC stage (*P* < 0.001, SHR = 1.760, and 95% CI 1.614-1.918) were significantly associated with cancer-specific survival as risk factors, while the surgery (*P* < 0.001, SHR = 0.295, and 95% CI 0.274-0.319), radiotherapy (*P* < 0.001, SHR = 0.808, and 95% CI 0.716-0.912), insurance (*P* < 0.001, SHR = 0.670, and 95% CI 0.567-0.791), and marriage (*P* < 0.001, SHR = 0.866, and 95% CI 0.809-0.927) were significantly associated with cancer-specific survival as protective factors.

From the Cox proportional hazards analysis of overall survival, age (*P* < 0.001, SHR = 1.009, and 95% CI 1.008-1.010), male (*P* < 0.001, SHR = 1.031, and 95% CI 1.014-1.049), black race (*P* < 0.001, SHR = 1.043, and 95% CI 1.021-1.065), high level of grade (*P* < 0.001, SHR = 1.036, and 95% CI 1.018-1.055), advanced AJCC stage (*P* < 0.001, SHR = 1.064, and 95% CI 1.043-1.085), surgery (*P* < 0.001, SHR = 0.808, and 95% CI 0.795-0.821), radiotherapy (*P* < 0.001, SHR = 0.932, and 95% CI 0.906-0.959), and marriage (*P* < 0.001, SHR = 0.949, and 95% CI 0.935-0.963) were significantly associated with OS as risk factors; while the other race (*P* < 0.001, SHR = 0.895, and 95% CI 0.877-0.914), surgery (*P* < 0.001, SHR = 0.808, and 95% CI 0.795-0.821), radiotherapy (*P* < 0.001, SHR = 0.932, and 95% CI 0.906-0.959), and marriage (*P* < 0.001, SHR = 0.949, and 95% CI 0.935-0.963) were associated with the OS as protective factors. It suggested that there was an association between therapy options (included surgery and radiotherapy) and better outcomes.

### 3.4. Development of the Nomograms

**Figure 2:**
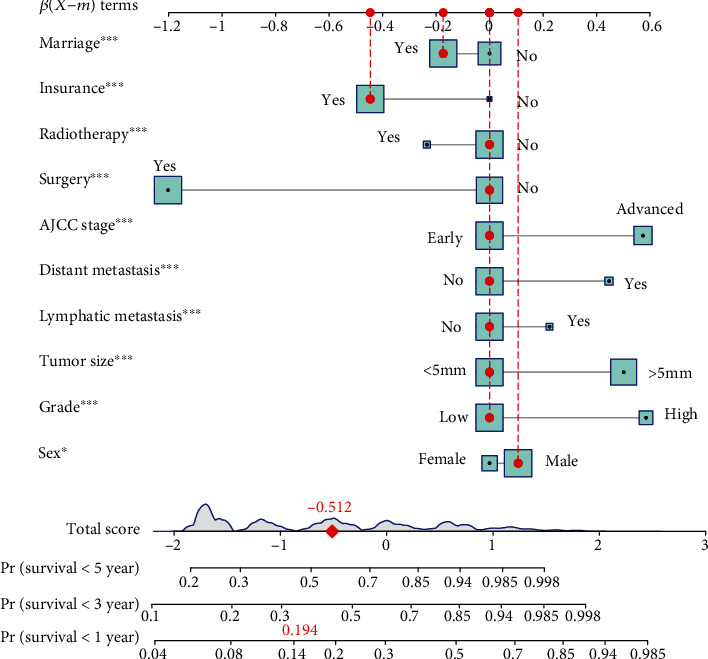
Nomogram to predict cancer-specific mortality of HCC patients. The item “Stage” in the figure represented the clinical stage; stages 1 and 2 were early, while stages 3 and 4 were advance, respectively. Tumor size is bounded by 5 mm. The item “Grade” represented the pathological grade; grades 1 and 2 were low, while grades 3 and 4 were high, respectively.

**Figure 3:**
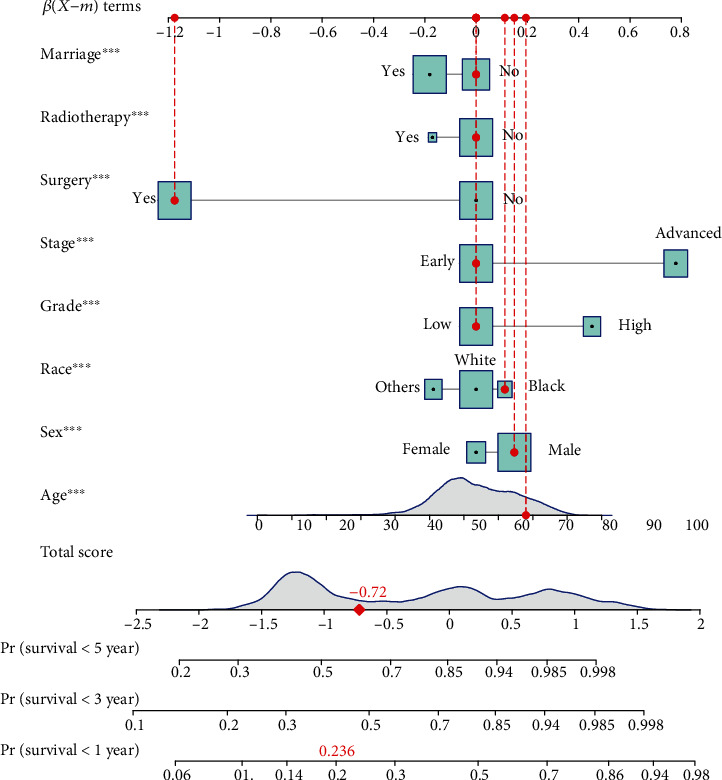
Nomogram to predict overall mortality of HCC patients. The item “Stage” in the figure represented the clinical stage; stages 1 and 2 were early, while stages 3 and 4 were advance, respectively. Tumor size is bounded by 5 mm. The item “Grade” represented the pathological grade; grades 1 and 2 were low, while grades 3 and 4 were high, respectively.

According to the results of univariable and multivariable analyses, marriage, insurance, radiotherapy, surgery, distant metastasis, lymphatic metastasis, tumor size, grade, sex, and the AJCC stage were selected as categories to develop the final cancer-specific prognostic nomogram, while the marriage, radiotherapy, surgery, AJCC stage, grade, race, sex, and age were selected to develop a nomogram for predicting overall mortality. The final nomogram to predict the cancer-specific mortality of HCC was developed and plotted in Figure 2, while the nomogram to predict the overall mortality of HCC was developed and plotted in Figure 3.

To predict the probability of mortality of HCC patients by utilizing the nomogram, we can find a certain score in each row of variables based on HCC patients' clinical characteristic and social status, and then, a straight line was draw up to the first line (points) to derive a risk score. Finally, we add all the risk scores and find the corresponding score in the row of total points; then, we could infer 1-, 3-, and 5-year mortality of HCC patients by drawing a straight line to the last 3 lines.

### 3.5. Evaluation of the Nomograms

**Figure 4:**
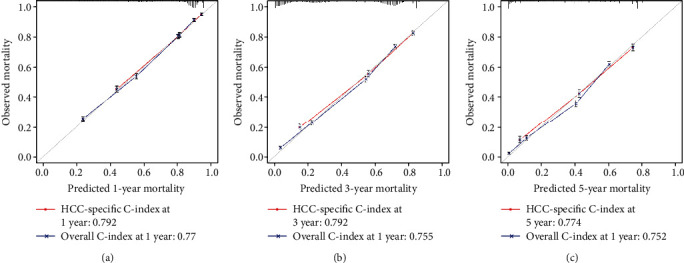
The calibration curve between predicted mortality and observed outcome. (a) The relationship between predicted 1-year mortality and observed mortality. (b) The relationship between predicted 3-year mortality and observed mortality. (c) The relationship between predicted 5-year mortality and observed mortality.

**Figure 5:**
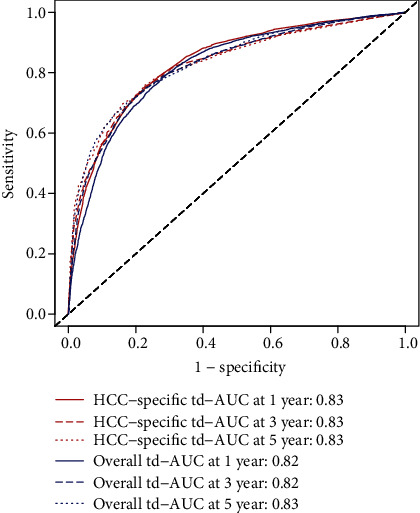
The area under the time-dependent receiver operating characteristic (ROC) curve (td-AUC).

The calibration curve and the ROC curve are plotted in Figures 4 and 5, respectively. The C-index of predicted 1-year cancer-specific mortality was 0.792, while the C-index of predicted 1-year overall mortality was 0.770 (Figure 4(a)); the C-index of predicted 3-year cancer-specific mortality was 0.776, while the C-index of predicted 3-year overall mortality was 0.755 (Figure 4(b)); the C-index of predicted 5-year cancer-specific mortality was 0.774, while the C-index of predicted 5-year overall mortality was 0.752 (Figure 4(c)). In Figure 5, the td-AUC values of predicted 1-, 3-, and 5-year cancer-specific mortality were 0.83, 0.83, and 0.83, respectively, while the td-AUC values of predicted 1-, 3-, and 5-year overall mortality were 0.82, 0.82, and 0.83, respectively. The calibration curve showed high consistency between the predicted mortality probability and the observed outcomes. Similarly, the AUC also reflected the predictive performance and reliability of the nomograms.

## 4. Discussion

Current guidelines from EASL and other relevant guidelines have indicated the necessity and significance of disease-specific risk stratification of HCC [1]. Fortunately, there are many disease-specific biomedical databases that are available to researchers to provide researchers with a foundation for developing nomograms and ensuring its prediction accuracy.

In the section of calculating the cumulative incidence of mortality, HCC patients who underwent surgical therapy possessed well survival rate compared with HCC patients without surgical therapy (Figure 1(b)), which indicated that surgical therapy is a significant protect factor to HCC. And it is similar to the univariable analysis, multivariable analysis, and competing risk analysis (Table 2). Additionally, the survival rate of patients is not obvious distinguish between patients with and without radiotherapy, especially in the early period (Figure 1(c)); however, it is a correlation between the radiotherapy and better observed outcomes. And we can also know that social status including insurance and marriage status have associated with better survival rate. Patients with insurance directly influence their quality of life. Raoof et al. [20] developed a tool for predicting quality of care to identify patients at a low level of quality, which suggested that insurance and marriage status can improve the survival rate by improving the quality care.

In these nomogram predictive models, age, sex, pathological grade, therapy, tumor size, T stage and M stage by AJCC, and social status were selected as input variables represented by a range of risk scores. Therefore, this all-around, personalized, acceptable, and graphical calculation means for determining the final risk score is utilized in the prediction of prognosis and survival rate. It has been evaluated by the C-index and td-AUC that the predictive performance of the nomogram model is worthy of recognition.

It has been reported that an individualized prediction is recognized as a crucial condition for the prognostic models [21]. The main purposes of this study were to predict cancer-specific mortality and the overall mortality for HCC patients, which is different from current published research related to predictive nomograms. In this study, we were mainly inclined to use long-term follow-up work for HCC patients. Our study is based on SEER data. Thanks to big data, the diagnosis of patients is accurately classified, eliminating the interference of their malignant tumor history. Moreover, the number of HCC patients recorded in the SEER database is huge, which helps us build a more accurate model. In addition, for doctors and patients, the items included in our nomogram are common clinically, easily accessible, and understandable items that can be easily carried out even in primary hospitals. After evaluation, the results of the C-index and AUC values indicate that our nomogram has a high predictive performance.

A work completed by Yang et al. involves the etiology and notes the relationship between the HCC and the chronic hepatitis B virus infection [22]. However, hepatitis B virus is not the only reason leading to the occurrence of HCC. Liu et al. addressed the diagnostic accuracy of HCC related to SCCA and SCCA-IgM and introduced a nomogram with moderate diagnostic accuracy which could provide a feasible and effective method for screening HCC [23]. Of course, there are several applications of nomograms in HCC that involved therapy [24], hepatotoxicity [24, 25], recurrence [26], metastasis [27], and microvascular invasion [28, 29].

There are also related researches about application for predicting the cancer-specific diseases. Song et al. [30] developed a predictive nomogram for predicting the survival of pancreatic cancer and considered to apply in clinical practice. Zhu et al. [31] utilized data from the SEER database to develop a predictive nomogram of Gleason score for prostate cancer to predict 5- and 10-year overall survival and cancer-specific survival. Li et al. [32] developed a nomogram for predicting overall survival and cancer-specific survival of adrenocortical cancer patients to help the clinicians make personal clinical therapy options. Zhou et al. [21] developed and evaluated a nomogram for the clinicians to predict survival of chondrosarcoma precisely and personally. Similarly, Zhong et al. constructed a nomogram to predict mortality probability of whole-stage small-cell lung cancer (SCLC). And Liu et al. [33] found that stage I SCLC can be managed scientifically by nomogram through Fine and Gray competing risk regression model. All of these above studies based on the bioinformatics database (such as SEER database) to develop a nomogram for many kinds of cancer to predict cancer-specific survival characteristics so that it can help clinicians make clinical decisions. In addition, nomogram represented as graph, which is utilized to communicate well between clinicians and patients. Nomogram is an intuitive, effective, and easily accepted tool for both clinicians and patients.

Limitations of this study include that our model lacked multicenter clinical samples for further validation to provide more convincing evidence. Moreover, the data we collected from the SEER database had a significant portion lacking full clinical information, resulting in data being wasted. Indicators such as alpha-fetoprotein and bilirubin were not included in this study, only recognized as “positive” or “negative” without exact lab values or a clearly stated standard to define “positive” and “negative”; therefore, they are excluded in this work. Additionally, the population from the SEER database were collected in the USA, but social factors and medical conditions vary from areas so that the conclusions are needed to be tested in other population.

## 5. Conclusions

We developed these nomograms to predict cancer-specific mortality and overall mortality, which will be helpful for clinicians to derive personal predictive information to identify whether a patient is at high risk of death. Then, the clinicians could give patient recommendation in time by utilizing the nomograms when combined with the actual disease condition to determine whether to adjust current therapy options that are beneficial to HCC patients. For patients with low predicted survival rate, combined with patient conditions, adjust the patient's expectation of prognosis and appropriately shorten the patient's follow-up interval and follow-up test items. That is the main significance of our study's findings, which can be effectively used to manage HCC patients and improve their quality of life.

## Figures and Tables

**Table 1:** Patients and survivor characteristics.

Characteristic	At diagnosis (*n* = 45,158)	Time after diagnosis		
		1 year (*n* = 24,647)	3 year (*n* = 16198)	5 year (*n* = 14218)
Age at diagnosis (years)				
≤64	26248 (58.125)	14999 (60.855)	10300 (63.588)	9205 (64.742)
65-79	14601 (32.333)	7818 (31.720)	4919 (30.368)	4203 (29.561)
≥80	4309 (9.542)	1830 (7.425)	979 (6.044)	810 (5.697)
Sex				
Male	34618 (76.660)	18614 (75.522)	12150 (75.009)	10628 (74.750)
Female	10540 (23.340)	6033 (24.478)	4048 (24.991)	3590 (25.250)
Race				
White	30737 (68.065)	16618 (67.424)	10798 (66.663)	9501 (66.824)
Black	5927 (13.125)	2904 (11.782)	1808 (11.162)	1562 (10.986)
Others	8285 (18.347)	4987 (20.234)	3483 (21.503)	3049 (21.445)
Unknown	209 (0.463)	138 (0.560)	109 (0.673)	106 (0.746)
Grade				
Grade 1 or 2	13432 (29.744)	8816 (35.769)	6188 (38.202)	5427 (38.170)
Grade 3 or 4	3773 (8.355)	1694 (6.873)	1103 (6.809)	960 (6.752)
Unknown	27953 (61.900)	14137 (57.358)	8907 (54.988)	7831 (55.078)
T category				
T0	32 (0.071)	12 (0.049)	6 (0.037)	5 (0.035)
T1	17916 (39.674)	12130 (49.215)	8720 (53.834)	7759 (54.572)
T2	9652 (21.374)	6606 (26.802)	4375 (27.010)	3842 (27.022)
T3	9378 (20.767)	3251 (13.190)	1712 (10.569)	1460 (10.269)
T4	1567 (3.470)	436 (1.769)	219 (1.352)	197 (1.386)
Unknown	6613 (14.644)	2212 (8.975)	1166 (7.198)	955 (6.717)
N category				
N0	35400 (78.391)	21405 (86.846)	14561 (89.894)	12857 (90.428)
N1	2737 (6.061)	816 (3.311)	403 (2.488)	351 (2.469)
Unknown	7021 (15.548)	2426 (9.843)	1234 (7.618)	1010 (7.104)
M category				
M0	35157 (77.853)	21967 (89.126)	14879 (91.857)	13109 (92.200)
M1	5568 (12.330)	1136 (4.609)	561 (3.463)	498 (3.503)
Unknown	4433 (9.817)	1544 (6.264)	758 (4.680)	611 (4.297)
Stage				
I	15357 (34.007)	11166 (45.304)	8213 (50.704)	7317 (51.463)
II	8157 (18.063)	5976 (24.246)	4064 (25.090)	3578 (25.165)
III	6739 (14.923)	2770 (11.239)	1508 (9.310)	1287 (9.052)
IV	7070 (15.656)	1725 (6.999)	846 (5.223)	746 (5.247)
Unknown	7835 (17.350)	3010 (12.212)	1567 (9.674)	1290 (9.073)
Surgery				
No	32291 (71.507)	13989 (56.757)	8064 (49.784)	7049 (49.578)
Yes	12464 (27.601)	10516 (42.666)	8064 (49.784)	7112 (50.021)
Unknown	403 (0.892)	142 (0.576)	70 (0.432)	57 (0.401)
Radiotherapy				
No	41894 (92.772)	23001 (93.322)	15185 (93.746)	13291 (93.480)
Yes	2820 (6.245)	1466 (5.948)	904 (5.581)	828 (5.824)
Unknown	444 (0.983)	180 (0.730)	109 (0.673)	99 (0.696)
Insurance				
No	1367 (3.027)	598 (2.426)	413 (2.550)	372 (2.616)
Yes	32083 (71.046)	18618 (75.539)	12835 (79.238)	11697 (82.269)
Unknown	11708 (25.927)	5431 (22.035)	2950 (18.212)	2149 (15.115)
Marriage				
No	19601 (43.405)	10044 (40.751)	6308 (38.943)	5492 (38.627)
Yes	23516 (52.075)	13454 (54.587)	9093 (56.137)	7997 (56.246)
Unknown	2041 (4.520)	1149 (4.662)	797 (4.920)	729 (5.127)

**Table 2:** Univariable and multivariable analyses of cancer-specific survival.

Cancer-specific survival (competing risk)				
Characteristic	Univariable		Multivariable	
	SHR (95% CI)	*P* value	SHR (95% CI)	*P* value
Age (years)	1.002 (1.001-1.003)	<0.001^∗^	1.000 (0.997-1.003)	0.986
Sex				
0 (female)	1		1	
1 (male)	1.154 (1.119-1.191)	<0.001^∗^	1.132 (1.046-1.225)	0.002^∗^
Race				
1 (white)	1		1	
2 (black)	1.183 (1.139-1.228)	<0.001^∗^	1.059 (0.964-1.164)	0.230
3 (others)	0.906 (0.876-0.938)	<0.001^∗^	0.959 (0.880-1.046)	0.345
Grade				
Low	1		1	
High	1854 (1.765-1.948)	<0.001^∗^	1.757 (1.631-1.892)	<0.001^∗^
Tumor size				
<5 mm	1		1	
≥5 mm	3.014 (2.926-3.105)	<0.001^∗^	1.577 (1.459-1.705)	<0.001^∗^
Lymphatic metastasis				
No	1		1	
Yes	2.001 (1.935-2.068)	<0.001^∗^	1.220 (1.087-1.368)	<0.001^∗^
Distant metastasis				
No	1		1	
Yes	3.559 (3.436-3.686)	<0.001^∗^	1.550 (1.398-1.718)	<0.001^∗^
Stage				
Early	1		1	
Advanced	3.651 (3.543-3.762)	<0.001^∗^	1.760 (1.614-1.918)	<0.001^∗^
Surgery (yes:1, no: 0)				
No	1		1	
Yes	0.263 (0.254-0.273)	<0.001^∗^	0.295 (0.274-0.319)	<0.001^∗^
Radiotherapy				
No	1		1	
Yes	1.286 (1.222-1.354)	<0.001^∗^	0.808 (0.716-0.912)	<0.001^∗^
Insurance				
No	1		1	
Yes	0.537 (0.501-0.576)	<0.001^∗^	0.670 (0.567-0.791)	<0.001^∗^
Marriage				
No	1		1	
Yes	0.825 (0.804-0.847)	<0.001^∗^	0.866 (0.809-0.927)	<0.001^∗^

HR: hazard ratio; CI: confidence interval. ^∗^*P* < 0.05.

**Table 3:** Univariable and multivariable analyses of overall survival.

Overall survival (Cox proportional hazards)				
Characteristic	Univariable		Multivariable	
	HR/Wald (95% CI)	*P* value	HR (95% CI)	*P* value
Age (years)	1.007 (1.007-1.007)	<0.001^∗^	1.009 (1.008-1.010)	<0.001^∗^
Sex				
0 (female)				
1 (male)	49.321 (0.886-0.934)	<0.001^∗^	1.031 (1.014-1.049)	<0.001^∗^
Race	314.874	<0.001^∗^		
1 (white)	2.475 (0.705-1.039)	0.116		
2 (black)	172.787 (1.185-1.258)	<0.001^∗^	1.043 (1.021-1.065)	<0.001^∗^
3 (others)	289.503 (1.357-1.469)	<0.001^∗^	0.895 (0.877-0.914)	<0.001^∗^
Grade	1225.702	<0.001^∗^		
Low	1141.247 (0.627-0.660)	<0.001^∗^		
High	6.827 (1.013-1.096)	0.009	1.036 (1.018-1.055)	<0.001^∗^
Tumor size	5717.775	<0.001^∗^		
<5 mm	4819.242 (0.339-0.360)	<0.001^∗^		
≥5 mm	369.677 (0.735-0.778)	<0.001^∗^	1.006 (0.989-1.023)	0.505
Lymphatic metastasis	3035.483	<0.001^∗^		
No	2055.399 (0.504-0.533)	<0.001^∗^		
Yes	43.073 (1.118-1.228)	<0.001^∗^	0.989 (0.958-1.022)	0.510
Distant metastasis	5667.517	<0.001^∗^		
No	1622.374 (0.478-0.512)	<0.001^∗^		
Yes	359.701 (1.440-1.566)	<0.001^∗^	1.006 (0.987-1.042)	0.316
Stage				
Early (1, 2)	3528.578 (0.394-0.418)	<0.001^∗^		
Advanced (3, 4)	134.646 (1.160-1.232)	<0.001^∗^	1.064 (1.043-1.085)	<0.001^∗^
Surgery (yes:1, no: 0)	6792.709	<0.001^∗^		
No	667.712 (3.726-4.623)	<0.001^∗^		
Yes	6733.661 (3.301-3.500)	<0.001^∗^	0.808 (0.795-0.821)	<0.001^∗^
Radiotherapy	117.182	<0.001^∗^		
No	60.740 (0.590-0.730)	<0.001^∗^		
Yes	17.389 (0.700-0.879)	<0.001^∗^	0.932 (0.906-0.959)	<0.001^∗^
Insurance	364.771	<0.001^∗^		
No	46.105 (1.174-1.338)	<0.001^∗^		
Yes	240.728 (0.806-0.846)	<0.001^∗^	0.961 (0.918-1.005)	0.081
Marriage	313.330	<0.001^∗^		
No	18.338 (1.069-1.195)	<0.001^∗^	1	
Yes	8.244 (0.872-0.974)	0.004^∗^	0.949 (0.935-0.963)	<0.001^∗^

HR: hazard ratio; CI: confidence interval. ^∗^*P* < 0.05.

## Data Availability

A total of 45,158 cases of available providing patient information and clinical characteristics were obtained from the SEER database during the period of 2004 to 2013. The SEER^∗^Stat software (Version 8.3.5, National Cancer Institute, Bethesda, MD, USA) was utilized to extract patient data with complete follow-up from the SEER database.
